# Multifunctional Properties of Nickel Nanoparticles Produced by Laser Ablation in Liquid

**DOI:** 10.3390/mi17080971

**Published:** 2026-08-17

**Authors:** Alexandru-Mihai Iamandi, Daniel-Liviu Ghiculescu, Gabriela Huminic, Angel Huminic, Ioan Mihail Ghițiu, Nicu Doinel Scărișoreanu

**Affiliations:** 1 National Institute for Laser, Plasma and Radiation Physics, 409 Atomistilor Street, 077125 Măgurele, Romania; alexandru.iamandi@inflpr.ro (A.-M.I.); ioan.ghitiu@inflpr.ro (I.M.G.); 2 National University of Science and Technology “Politehnica”, Splaiul Independentei No. 313, 060042 Bucharest, Romania; daniel.ghiculescu@gmail.com; 3 Mechanical Engineering Department, Transilvania University of Brasov, 29, Bulevardul Eroilor, 500036 Brasov, Romania; gabi.p@unitbv.ro (G.H.); angel.h@unitbv.ro (A.H.); 4 Faculty of Physics, University of Bucharest, Street Atomiștilor 405, 077125 Măgurele, Romania

**Keywords:** nanoparticles, laser ablation, photoelectrochemical water splitting, heat conductivity, viscosity, thermal performance

## Abstract

This study explores the multifunctional properties of Ni nanoparticles obtained by laser ablation in liquid, with emphasis on the potential use of these nanoparticles in different applications such as cooling fluids or photoelectrochemical ones. The Ni nanoparticles were synthesized by the laser ablation in liquid technique using an Nd-YAG laser and ultrapure water as liquid. The structural, dimensional, morphologic, and stoichiometric characterizations of the nanoparticles were performed using different techniques such as transmission electron microscopy (TEM), energy dispersive X-ray (EDS) and dynamic light scattering spectroscopy (DLS). Nickel nanoparticles with sizes ranging from 5 to 15 nm in diameter were obtained. The experimental measurements were performed to determine the thermal conductivity and viscosity of the obtained nanofluids, essential parameters in the evaluation of the cooling fluid performances. Loading TiO_2_ thin films with Ni nanoparticles led to the enhancement of the photoelectrochemical water splitting properties of TiO_2_ thin films, the Ni nanoparticles acting on the collecting, transferring and separating the photogenerated charges and ultimately improving the overall anodic and cathodic efficiencies. The results obtained can contribute to the development of innovative, multifunctional solutions based on non-precious metals for cooling and water splitting systems used in industrial, electronics and other applications.

## 1. Introduction

The increasing demand for multifunctional nanomaterials for energy conversion, thermal management, and environmental applications has stimulated extensive research on nanoparticle-based systems [1,2,3,4,5,6,7,8,9,10,11]. For example, high-efficiency thermal management in automotive engines, electronic cooling, solar collectors, and industrial heat exchangers has led to extensive research on nanofluids—engineered suspensions of nanoparticles (typically < 100 nm) dispersed in conventional base fluids such as water, ethylene glycol, or oils. Nanofluids have been widely investigated for their potential to enhance thermal conductivity and convective heat transfer beyond that of conventional coolants [1,2]. Comprehensive reviews found in the literature [3,4,5] describe the mechanisms responsible for heat transfer enhancement, including Brownian motion, interfacial liquid layering, and particle clustering. Among the wide range of nanoparticles studied, metallic elements (nickel (Ni) or oxides of transition metallic elements, such as Fe_2_O_3_, titanium dioxide (TiO_2_), or others) have emerged as particularly promising candidates due to their favorable thermophysical properties, availability, and tuneable particle size.

The thermal performance of nanofluids strongly depends on the type, size, concentration, and stability of the dispersed nanoparticles. Metallic nanoparticles (e.g., Ag, Cu, and Ni) generally provide higher thermal conductivity enhancement, whereas metal oxide nanoparticles (e.g., TiO_2_, Al_2_O_3_, and Fe_2_O_3_) are widely used because of their good chemical stability, low cost, and ease of synthesis [3,4,5,6,9]. Among oxide materials, TiO_2_ has been extensively investigated owing to its excellent stability and dispersion characteristics. Primary particle sizes reported in nanofluid studies generally range between 10 and 100 nm, while experimental investigations commonly employ TiO_2_ nanoparticles of 15–50 nm, with representative studies reporting average particle sizes of approximately 21 nm [12,13,14,15]. TiO_2_-based nanofluids have demonstrated improved heat transfer (by approximately 6–11% at low nanoparticle concentration 0.2–1.2%), although the overall performance depends on nanoparticle concentration, dispersion stability, viscosity, and operating conditions [7,13,14,16,17,18].

Compared with other widely investigated nanoparticles such as Al_2_O_3_ and CuO, the TiO_2_ nanoparticles generally offer superior chemical stability and lower cost, although CuO and metallic nanoparticles often yield higher thermal conductivity enhancement at equivalent concentrations [18,19]. Overall cooling performance therefore depends not only on particle material but also on diameter (typically 10–80 nm in most studies), concentration, surface chemistry, and flow regime. Optimization requires balancing thermal conductivity improvement with viscosity increase, dispersion stability, and long-term reliability [2,7].

Related to the metallic nanoparticles, silver (Ag) and copper (Cu) nanoparticles are often reported to provide the highest thermal conductivity enhancement due to their very high intrinsic thermal conductivity. However, nickel (Ni) nanoparticles represent an attractive alternative because they combine relatively high thermal conductivity with additional properties such as magnetic behavior, catalytic activity, and good mechanical stability. The thermal conductivity of nickel has been reported to be significantly higher than that of alumina, with values typically around 70–90 W·m^−1^·K^−1^ for Ni, compared with approximately 20–35 W·m^−1^·K^−1^ for Al_2_O_3_, depending on the material purity [3,5,7]. Because of this difference, nanofluids containing metallic nanoparticles such as Ni can often produce stronger thermal conductivity enhancement than oxide-based nanofluids at similar concentrations.

Nickel nanoparticles have emerged as particularly attractive nanomaterials because they combine relatively high thermal conductivity with magnetic, catalytic, and electrical properties. Their large specific surface area enhances catalytic activity, while their ferromagnetic behavior enables magnetic manipulation and catalyst recovery in fluid systems [8,9,10,11]. In addition, Ni nanoparticles have been investigated for applications in thermal management, catalysis, energy conversion, sensing, and advanced coatings [3,4]. Compared with oxide nanoparticles, Ni nanoparticles can provide greater thermal conductivity enhancement in nanofluids, although their practical implementation requires careful control of oxidation, dispersion stability, and viscosity [3,4,5,6,7,18,20,21]. Table 1 summarizes their main properties together with representative particle sizes reported in the literature. This comparison highlights the unique multifunctional characteristics of Ni nanoparticles compared with other commonly used nanomaterials.

Beyond thermal management, nickel nanoparticles have attracted considerable attention as cocatalysts for TiO_2_-based photocatalytic and photoelectrochemical (PEC) systems. Although TiO_2_ is one of the most widely studied photocatalysts because of its chemical stability and low cost, its wide band gap and rapid electron–hole recombination significantly limit its efficiency. Recent studies have demonstrated that Ni modification enhances visible-light absorption, improves charge separation, and facilitates interfacial electron transfer, resulting in enhanced photocatalytic and PEC performance [23,24,25,26,27,28,29].

In this study, nickel nanoparticles synthesized by pulsed laser ablation in liquid (PLAL) were investigated as multifunctional nanomaterials for two complementary applications. PLAL represents an environmentally friendly alternative capable of producing chemically clean nanoparticles directly in ultrapure water without chemical additives. Compared with conventional chemical synthesis, this technique eliminates the need for chemical precursors and stabilizing agents, resulting in high-purity, surfactant-free nanoparticles with clean surfaces and minimal contamination. These characteristics are particularly advantageous for catalytic applications, where accessible active surface sites are essential, as well as for heat-transfer applications by preserving the intrinsic properties of the nanoparticles. Furthermore, PLAL enables control of nanoparticle characteristics through laser processing parameters such as wavelength, pulse energy, and liquid volume. First, their potential as nanofluid additives were evaluated through thermal conductivity, viscosity, and cooling performance measurements. Second, their cocatalytic activity was investigated by modifying TiO_2_ photoelectrodes for PEC applications. By combining these two research directions, this work demonstrates the multifunctional potential of laser-generated Ni nanoparticles and provides insight into their applicability for both advanced thermal management and energy-related technologies.

## 2. Experimental Preparation of Nanofluids

**Figure 1:**
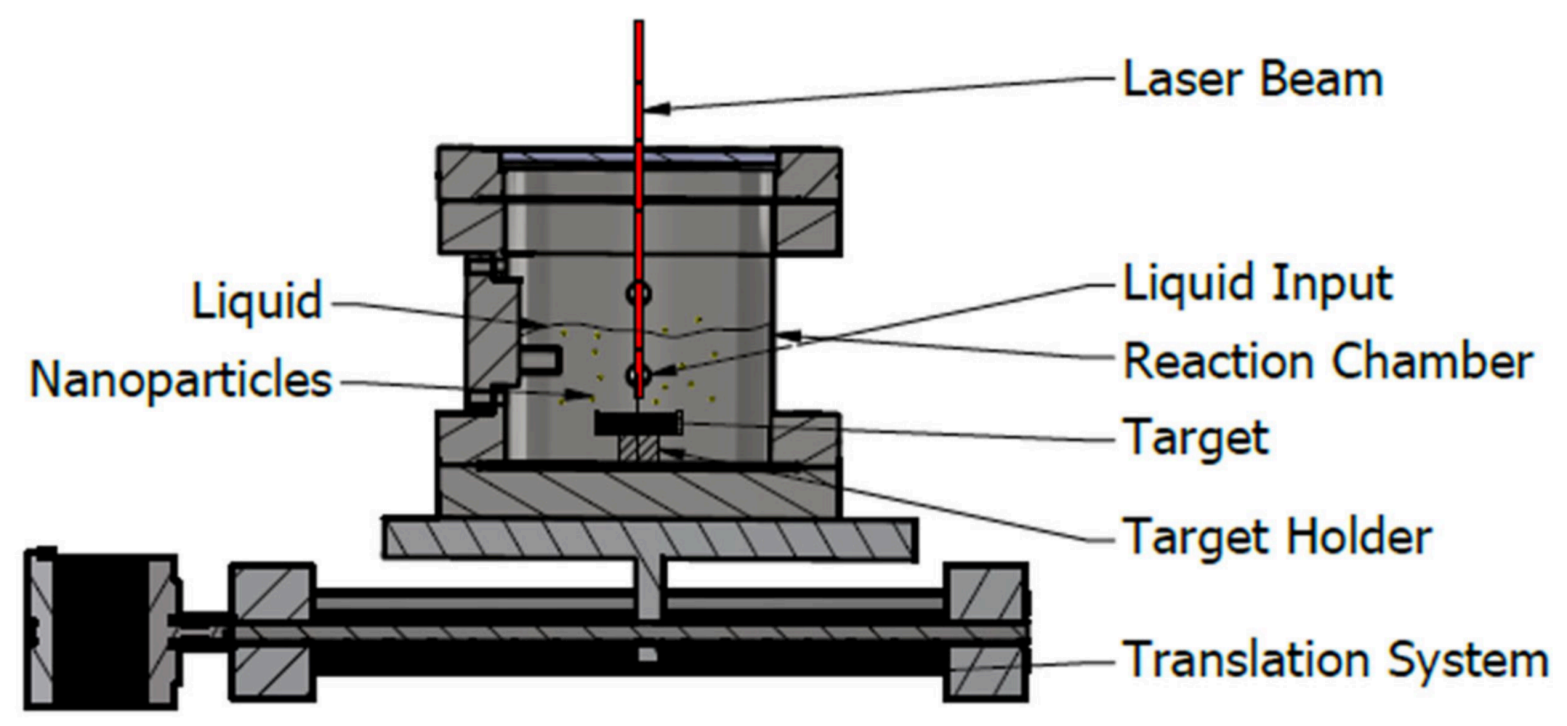
The experimental setup.

The laser ablation process was performed using a Continuum Surelite II laser (Amplitude Laser Inc., Milpitas, CA, USA) at different wavelengths (1064–532 nm). The nickel target was placed at the bottom of the reaction chamber (Figure 1) on a holder, positioned 2.5 cm above the chamber base. Mirrors arranged in the optical path directed the laser beam onto the target surface, with an energy range of approximately 350–700 mJ. The laser beam produced an irradiated spot with a diameter of approximately 4.7 mm. To enable laser beam scanning, the reaction chamber was mounted on a translation stage for X-axis movement, while adjustable mounts controlled scanning along the Y-axis. The laser beam was scanned over a rectangular area of 12 × 22 mm (X × Y), corresponding to a total scanning area of 264 mm^2^. The nickel target was fully immersed in ultrapure water (UPW).

This study investigates the thermal properties (thermal conductivity and viscosity) of base fluid and nanofluids using advanced measurement techniques. For comparison purposes, Ti nanoparticles were also produced by PLAL using a commercial 1-inch Ti target (99.9% purity, Kurt J. Lesker, Jefferson Hills, PA, USA). The target was immersed in the liquid medium and irradiated with a pulsed laser under the experimental conditions described below. A pulsed laser was employed because its high peak power enables efficient ablation of the target in the liquid environment while minimizing thermal effects and contamination, using the following experimental conditions. The thermal conductivity of the samples was measured using the KD2-Pro Thermal Property Analyzer (Decagon Devices, Inc., Pullman, WA, USA), while the viscosity was evaluated using the Brookfield Programmable Viscometer (Ametek Brookfield, Middleborough, MA, USA). Both properties were measured under controlled temperature conditions, maintained by a thermostatic bath (Labo S200-H13), with an accuracy of ±0.03 °C. Several measurements were performed at various temperatures to evaluate the effects of temperature on both thermal conductivity and viscosity. The arithmetic mean of these measurements was calculated and used for analysis to ensure reliability and consistency in the results. The nanoparticle concentration was determined gravimetrically. Each glass substrate was dried and weighed prior to sample deposition using an analytical balance. Subsequently, 5 mL of the colloidal nanoparticle suspension was deposited onto the substrate by the drop-casting method and allowed to dry completely until complete solvent evaporation. The substrate was then weighed again, and the mass difference corresponding to the deposited nanoparticles was calculated. The nanoparticle concentration (Table 2) was determined by dividing the deposited nanoparticle mass by the deposited suspension volume (5 mL).

The concentration was calculated according to the following equation:

(1) $$C\;=\;\frac{M_{f}-M_{i}}{V},$$

where *M_f_* is the mass after solvent evaporation and *M_i_* is the initial mass of the glass substrate and *V* is the volume of the deposited colloidal suspension (5 mL).

High-resolution transmission electron microscopy analysis was carried out with a ThermoFisher Spectra 300 S/TEM to examine particle morphology and structural features. The hydrodynamic size distribution and stability of the nanoparticles were evaluated using a HORIBA SZ-100 DLS system.

Additionally, their PEC performance was assessed with an Autolab PGSTAT302N, providing insight into their charge-transfer behavior and cocatalytic activity. The PEC measurements were made in a three-electrode configuration system in a quartz cell. For the working electrode to act as photoactive material, TiO_2_ thin films were specially deposited by Pulsed Laser Deposition on Pt/SiO_2_/Si substrates, using a Nd-YAG laser (266 nm) working at 10 Hz repetition rate. The counter electrode was a Pt wire, and the reference electrode was Ag/AgCl (3.5 M KCl). The electrical connections were made on the Pt substrates using conductive wire and silver paste. The samples were electrically isolated with a non-corrosive epoxy resin, to avoid electrical short circuit of the substrate in contact to electrolyte solutions. The measurements were performed in 0.5 M NaOH solution (pH = 13.7). The laser diode used for irradiation emits at 405 nm (5 mW output power). To avoid the effect of concentration polarization, a small scanning rate (5 mV/s) was used for potentiodynamic measurements.

## 3. Results and Discussion

### 3.1. Morphological, Structural and Compositional Properties of Ni Nanoparticles

**Figure 2:**
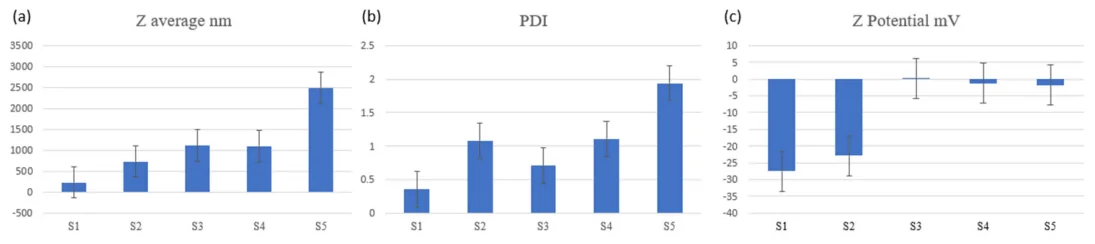
(**a**) DLS Z-average data. (**b**) Polydispersity index data. (**c**) Zeta potential data.

Colloidal solutions and nanoparticles are characterized using DLS (Figure 2). In this technique, a laser beam passes through the colloidal solution, and the scattered light is measured. By analyzing the fluctuations in the intensity of the scattered light over time, the particle size can be determined. The Z-average represents the average hydrodynamic diameter of the nanoparticles measured by DLS. The polydispersity index (PDI) ranges from 0 to 1, with 0 indicating a completely homogeneous population and 1 signifying a highly heterogeneous one [30,31,32]. The magnitude of the zeta potential reflects particle stability, a higher absolute value indicates greater stability because of stronger electrostatic repulsion between particles [30,31,32].

In the graphs presented below, you can see the values obtained through DLS characterization of the samples studied.

A significant variation in nanoparticle size is observed between samples. The S1 probe has the smallest particles (~350 nm), and S5 the largest (>2500 nm). This indicates that samples made using higher energy and lower liquid volume (670 mJ, 400 mL UPW) have a narrower size distribution than samples made using lower energy and higher liquid volume (440 mJ, 800 mL UPW).

The PDI ranges from 0.4 (S1/mL probe) to over 2.0 (S5). Values greater than 0.7 indicate a broad particle size distribution, so samples S2 and S5 are the most heterogeneous. The S1 probe has the most uniform particle population.

Only samples with S1 and S2 show significant negative zeta potential (below −20 mV), indicating increased colloidal stability. The rest of the samples have values close to zero, which may lead to rapid aggregation.

The HR-TEM techniques were employed to investigate the morphological characteristics and size distribution of the synthesized nanoparticles. Representative TEM images and the corresponding particle size distribution histograms for various samples are presented in Table 3. The particle size distribution histogram was generated by manually measuring the diameters of 20 representative nanoparticles from the HAADF-STEM images using ImageJ software 1.54p.

The TEM images reveal notable differences in particle morphology and aggregation behavior among the analyzed samples. The sample S1 TEM image displays a relatively dense aggregation of nanoparticles with varied shapes and sizes with an average length of 7.14 nm, suggesting a heterogeneous population. The corresponding histogram supports this observation, showing a broad distribution with a standard deviation (SD) of 0.24, indicating considerable polydispersity.

Similarly, the image of probe S2 exhibits partially dispersed nanoparticles, with average size of 7.34 nm and with more spherical morphology. However, the particle size distribution remains broad (SD = 0.25), further confirming significant variation in particle dimensions.

In contrast, the samples S3 and S5 display improved dispersion and more uniform particle morphologies. The sample S3 image, for instance, shows moderately well-dispersed particles with a standard deviation of 0.22 and an average size of 7.70 nm, while the S4 sample exhibits a slightly better uniformity (SD = 0.22) with an average length of 11.30 nm. Notably, the image of probe S5 shows highly monodisperse nanoparticles with minimal aggregation. This is corroborated by the narrowest size distribution observed among all samples, with a standard deviation of only 0.15 and an average length of 4.70 nm.

The particle size distributions were plotted based on measurements of the nanoparticle lengths (in nm) obtained from multiple TEM fields, and each histogram includes a Lognormal distribution curve to highlight the spread of particle sizes. These results suggest that the synthesis conditions used for the bottom-right sample were optimal in producing uniform, well-dispersed nanoparticles with reduced polydispersity.

High-resolution TEM analysis confirms that the sample consists of nickel-based nanoparticles exhibiting a core–shell morphology. The HAADF imaging reveals well-defined spherical particles with strong contrast. Elemental mapping shows a distinct distribution: nickel (Ni) is predominantly localized in the cores of the particles, while oxygen (O) is concentrated at or near the particle surfaces. This spatial arrangement suggests the formation of a nickel oxide (NiO) shell surrounding a metallic nickel core. The observed structure likely results from surface oxidation of the nanoparticles and may offer advantageous properties for applications in catalysis, energy storage, and related nanomaterial technologies.

**Figure 3:**
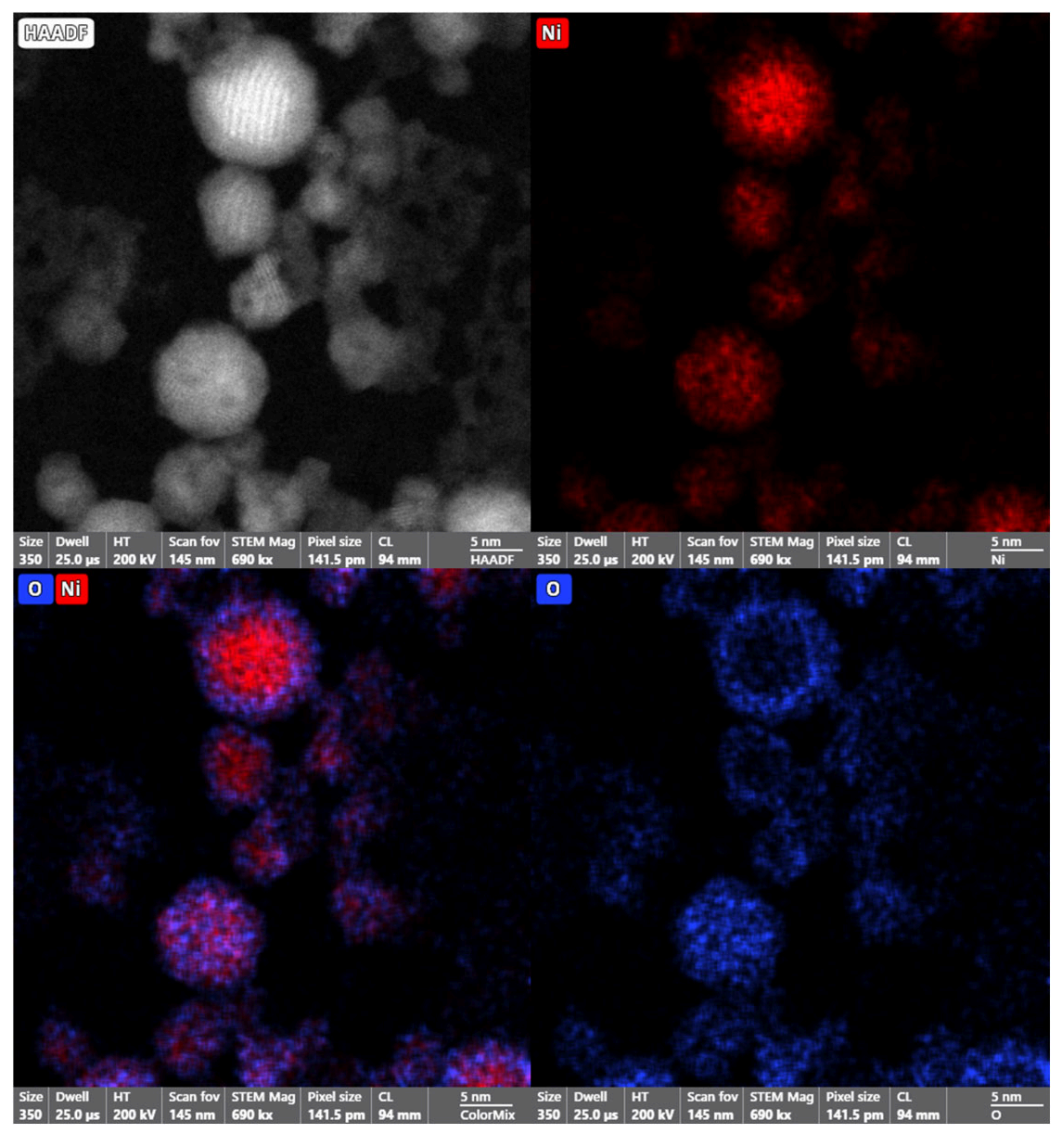
TEM images of Ni nanoparticles of sample S3.

In Figure 3, the STEM image, together with the corresponding EDS elemental maps, confirm the presence of both Ni and O within the synthesized nanoparticles. The simultaneous detection of oxygen suggests partial surface oxidation of the nanoparticles, resulting in Ni-based nanoparticles with a thin NiO surface layer. Such partial oxidation is commonly reported for Ni nanoparticles synthesized by pulsed laser ablation in water due to their high surface reactivity and exposure to dissolved oxygen immediately after synthesis [31,33].

Calculated interplanar spacing and lattice constants are shown in Table 4. The d-spacing and lattice constants were very close to values obtained by [34].

**Figure 4:**
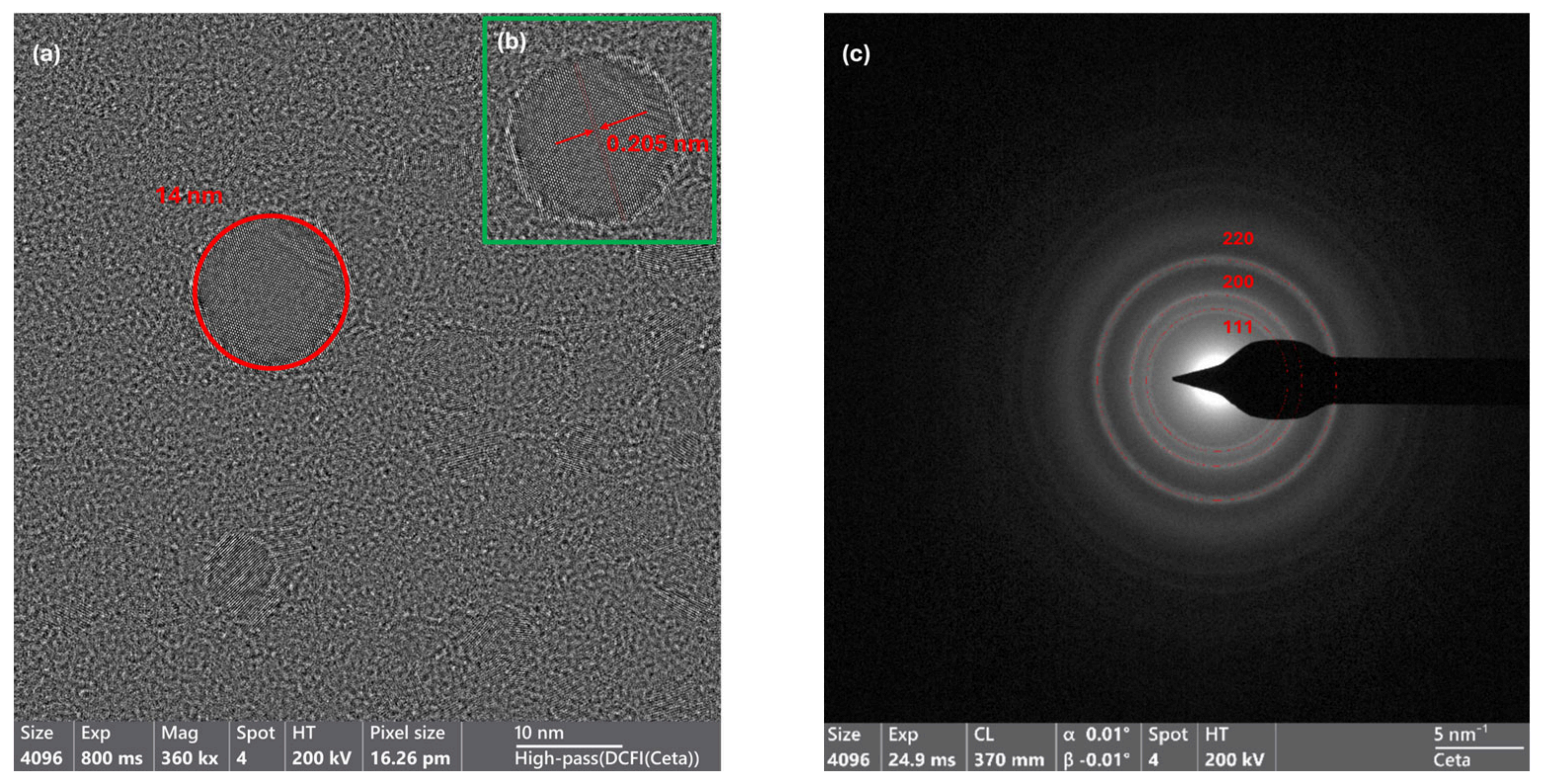
(**a**) Bright-field TEM image of Ni-based nanoparticles—sample S1. (**b**) High-resolution TEM image of single nickel-based particle and electron diffraction pattern (upper inset of Figure 4b) shows crystalline nature of the nanoparticles. (**c**) High-resolution TEM image of crystalline Ni nanoparticles and corresponding electron diffraction pattern (lower inset of Figure 4c).

In Figure 4, the high-resolution transmission electron microscopy (HRTEM) analysis is presented to study the structural and crystallographic properties of synthesized Ni-based nanoparticles. Figure 4a is a high-resolution TEM image showing a nearly spherical nanoparticle. The red circle highlights a single nanoparticle with a measured area of 187.723 nm^2^ and a diameter of approximately 15.5 nm, indicating relatively large nanoparticle size for high-resolution imaging. The lattice fringes visible suggest good crystallinity. Figure 4b shows the measured interplanar spacing of 0.205 nm, which is in good agreement with the reported value of approximately 0.209 nm for the (200) plane of face-centered cubic (FCC) NiO, indicating the presence of surface-oxidized Ni-based nanoparticles [34].

In Figure 4c, the diffraction rings are clearly indexed as (111), (200), and (220), which match the standard FCC structure of nickel oxide [34].

### 3.2. Thermal Conductivity and Dynamic Viscosity of Ni Nanoparticles-Based Nanofluids

This study investigates the thermal properties (thermal conductivity and viscosity) of base fluid and nanofluids using advanced measurement techniques. For the evaluation of thermal properties of the Ni samples, several measurements were performed at various temperatures to evaluate the effects of temperature on both thermal conductivity and viscosity. The arithmetic mean of these measurements was calculated and used for analysis to ensure reliability and consistency in the results.

**Figure 5:**
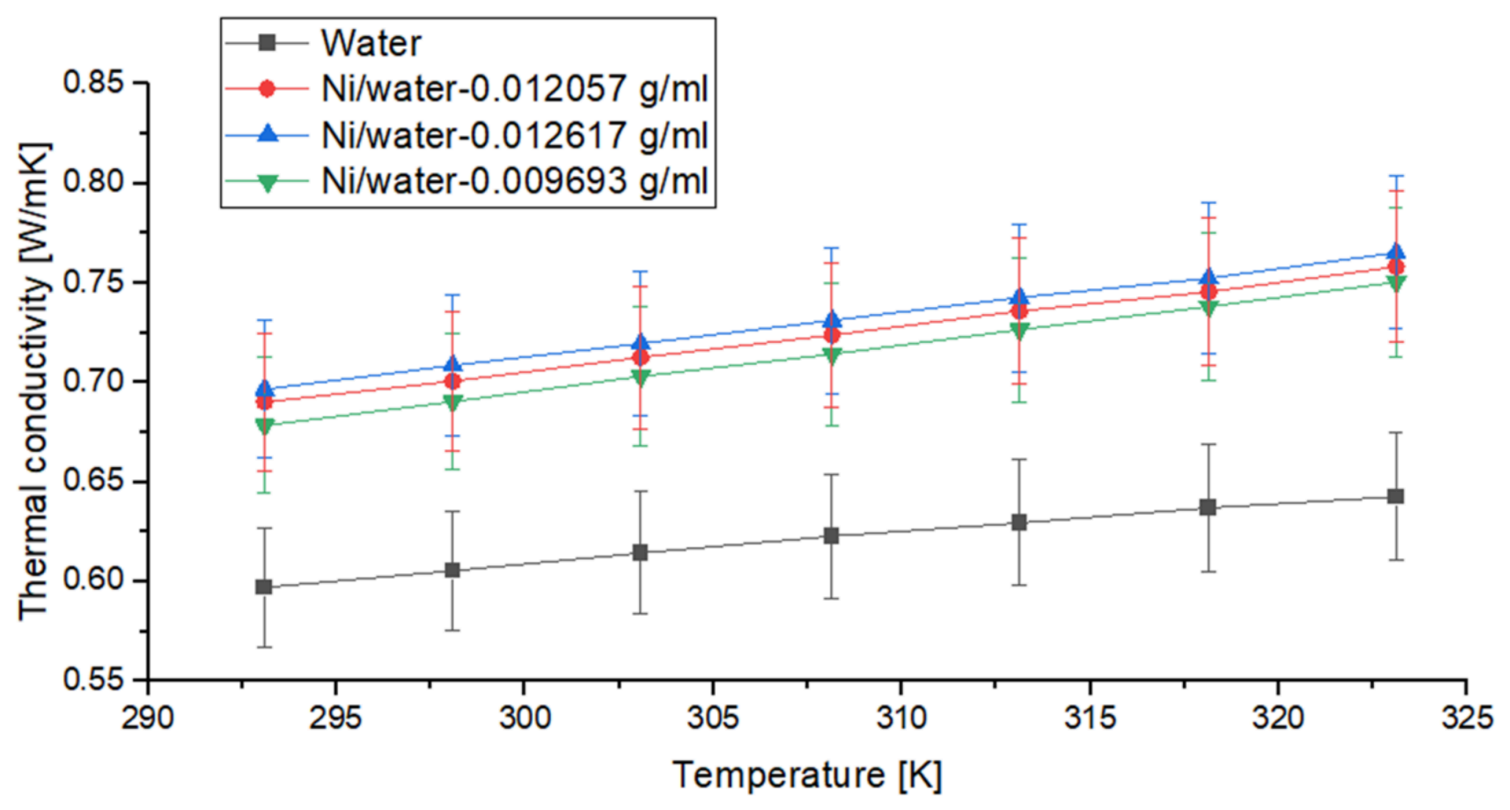
The variation in thermal conductivity with temperature.

Figure 5 illustrates the variation in thermal conductivity with temperature for water and nickel (Ni) nanoparticle-based nanofluids at different concentrations (of S1, S3, and S3). As temperature increases, the thermal conductivity of all samples follows an upward trend, indicating improved thermal conductivity at higher temperatures.

The base fluid exhibits the lowest thermal conductivity across the examined temperature range. In contrast, the Ni/water nanofluids show an improvement in thermal conductivity. Furthermore, the results indicate a positive correlation between nanoparticle concentration and thermal conductivity, the higher concentrations yielding superior thermal performance. The most significant enhancement is observed in the Ni/water nanofluid with the highest concentration (S1), which consistently outperforms the lower concentrations and pure water. This behavior can be attributed to the enhanced thermal transport mechanisms facilitated by suspended nanoparticles, including increased Brownian motion and improved thermal interactions within the fluid matrix. Also, this trend highlights the potential of Ni/water nanofluids as an effective medium for improving thermal performance in heat transfer applications.

**Figure 6:**
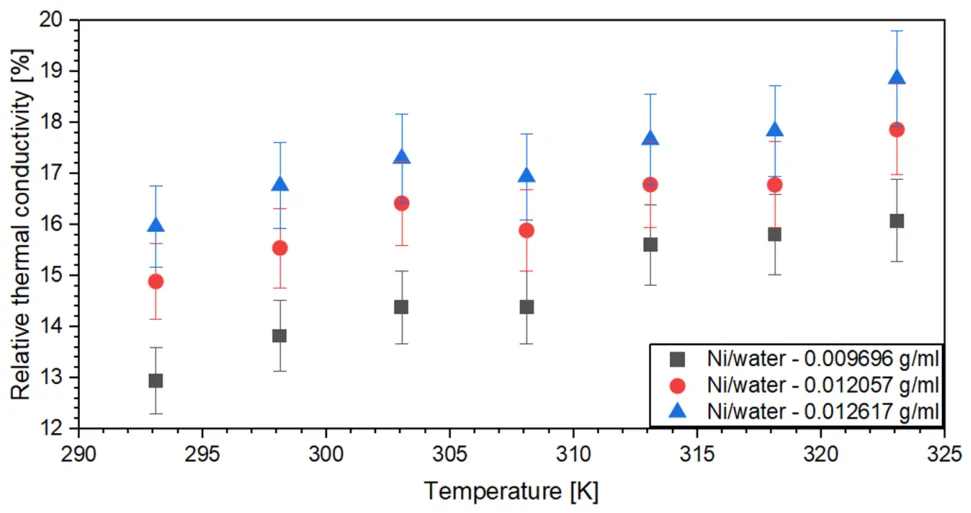
Relative thermal conductivity as a function of temperature.

Figure 6 presents the relative thermal conductivity, defined as:

(2) $$\left[\frac{K_{nf}-k_{bf}}{k_{bf}}\right]\cdot 100,$$

where k_nf_ represents thermal conductivity of the fluid with nanoparticles and k_bf_ represents the thermal conductivity of the base fluid. The calculated value represents the percentage enhancement in thermal conductivity of the nanoparticles suspension relative to the base fluid.

With temperature for Ni/water nanofluids at different nickel nanoparticle concentrations, the results demonstrate a clear trend in which the relative thermal conductivity increases with rising temperature, indicating an improvement in the heat transfer capability of the nanofluid.

Among the tested concentrations, the nanofluid with the highest Ni content (S1) exhibits the most significant enhancement in thermal conductivity. This is followed by the nanofluid containing S3, while the lowest concentration (S2) results in the least improvement. At lower temperatures (293 K), the relative thermal conductivity enhancement ranges from approximately 12% to 16%, depending on the nanoparticle concentration. As the temperature increases (323 K), the enhancement further rises, reaching values between 15% and 19%. This trend indicates that the effectiveness of Ni nanoparticles in augmenting thermal conductivity becomes more pronounced at elevated temperatures, which may be attributed to increased nanoparticle mobility and enhanced heat transfer mechanisms.

**Figure 7:**
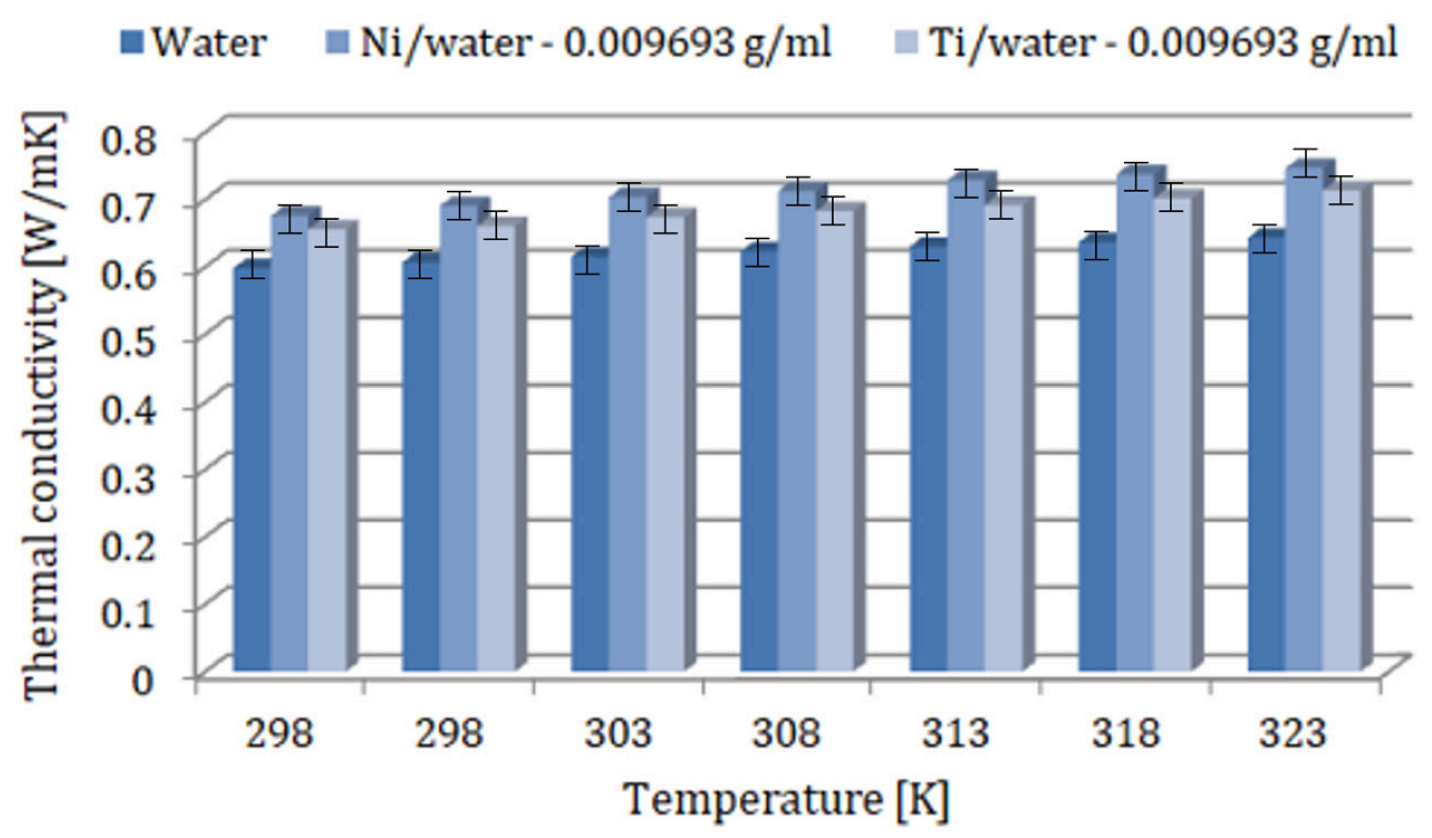
The thermal conductivity for varying temperatures of water, nickel-based nanofluid and titanium-based nanofluid.

Only for comparison purposes, Ti nanoparticles were also prepared in the same PLAL conditions as the Ni nanoparticles and were tested for their thermal properties. The thermal conductivity for varying temperatures of pure water, nickel-based nanofluid (Ni/water), and titanium-based nanofluid (Ti/water), each at a concentration of S3, is depicted in Figure 7. The results reveal a clear trend where the thermal conductivity of all three fluids increases with temperature, suggesting an improvement in thermal conductivity at higher temperatures.

**Figure 8:**
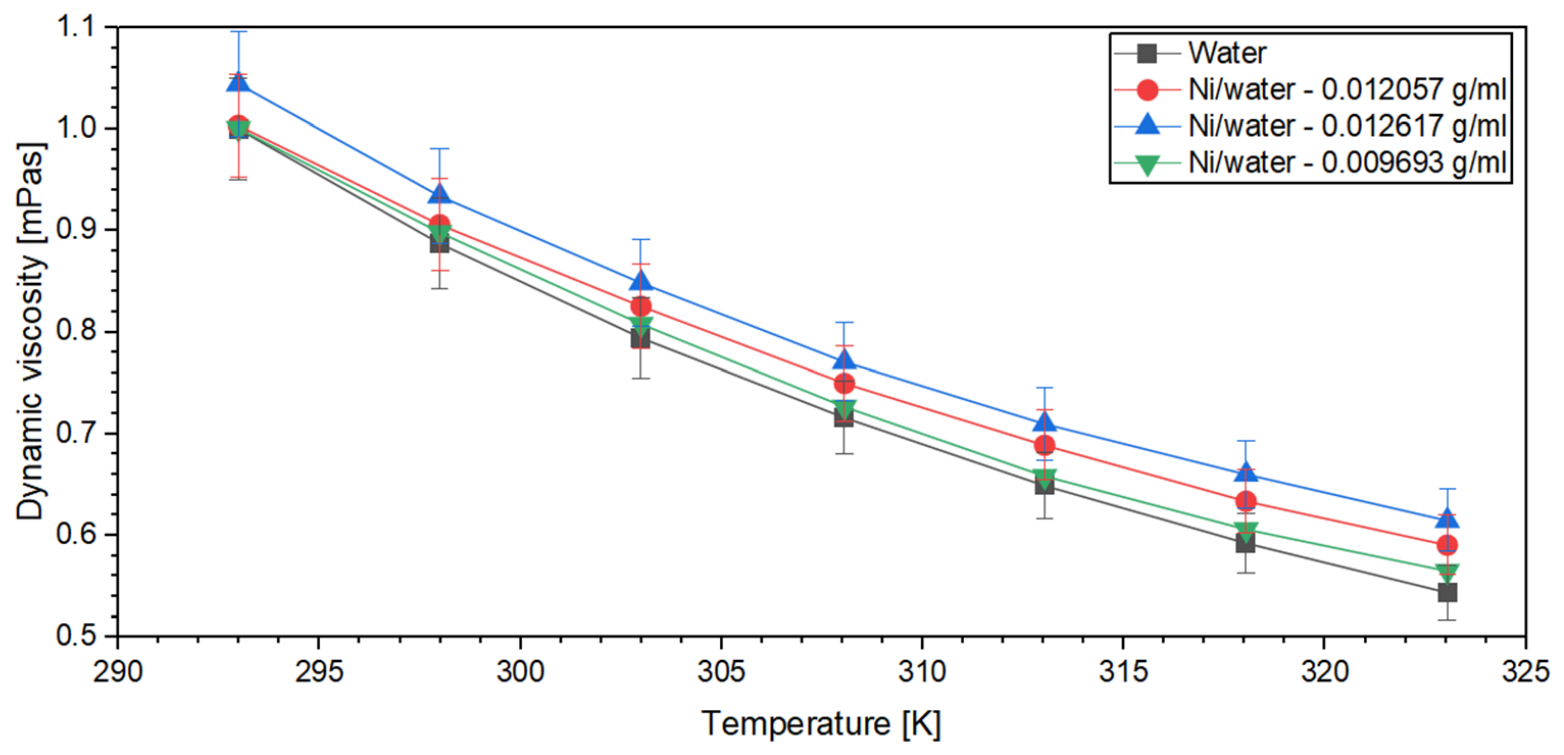
The variation in dynamic viscosity as a function of temperature.

Moreover, both nanofluids demonstrate improved thermal conductivity compared to pure water, but Ni/water consistently exhibits higher thermal conductivity than Ti/water at every temperature. As temperature increases from 298 K to 323 K, the thermal conductivity of both nanofluids rises, indicating that their heat transfer efficiency improves with temperature. However, the Ni/water nanofluid exhibited a higher thermal conductivity than the TiO_2_/water nanofluid throughout the investigated temperature range. Compared with pure water, the thermal conductivity enhancement was approximately 15–18% for the Ni/water nanofluid, whereas the TiO_2_/water nanofluid showed an enhancement of approximately 10–13%, suggesting that nickel nanoparticles contribute more effectively to thermal enhancement at the same concentration. Several factors could explain this performance difference. Ni nanoparticles have a higher intrinsic thermal conductivity than Ti nanoparticles, allowing for more efficient heat transfer within the fluid. Additionally, Ni nanoparticles may disperse more effectively in water, promoting better particle–fluid interactions and enhancing heat transfer mechanisms such as nano-convection of fluid around the particles due to their motion [16,35]. This improved dispersion likely contributes to the greater thermal conductivity observed in Ni-based nanofluid compared to Ti-based nanofluid. Figure 8 illustrates the variation in dynamic viscosity as a function of temperature for both water and nickel (Ni) nanoparticle-based nanofluids at different concentrations. The viscosity measurements show that the Ni/water nanofluid exhibited an increase in viscosity compared to the base fluid (pure water). This increase in viscosity became more pronounced with higher nanoparticle concentrations, indicating that the addition of nanoparticles introduces resistance to flow. The increase in viscosity is likely due to the interaction between the nanoparticles and the base fluid molecules, which may lead to the formation of nanoparticle clusters or a more complex network structure within the fluid [36,37]. Interestingly, the viscosity did not exhibit a strictly proportional increase with nanoparticle concentration under the investigated conditions, which may be associated with nanoparticle–fluid interactions. This non-linear behavior could be attributed to factors such as nanoparticle agglomeration [36], changes in the microstructure of the nanofluid, or alterations in the rheological behavior at higher concentrations. As observed in Figure 8, viscosity decreases with increasing temperature, which is consistent with the general behavior of most fluids. The thermal energy from the rising temperature reduces the internal friction between the fluid molecules, allowing them to flow more easily [38].

**Figure 9:**
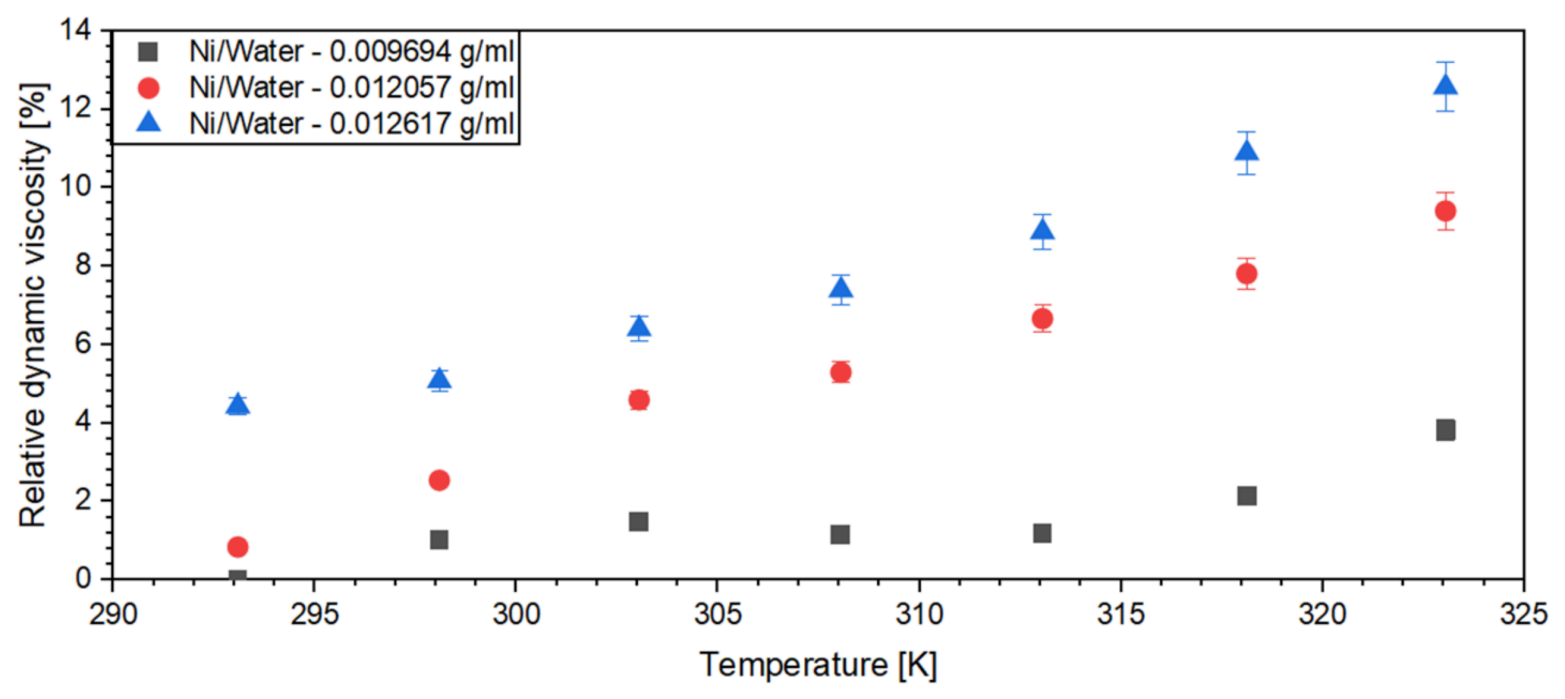
Relative dynamic viscosity as a function of temperature.

Figure 9 presents the relative dynamic viscosity, defined as:

(3) $$\left[\frac{\mu_{nf}-\mu_{bf}}{\mu_{bf}}\right]\cdot 100$$

where *μ_nf_* is the dynamic viscosity of the nanofluid and *μ_bf_* is the dynamic viscosity of the base fluid. The calculated value represents the percentage change in the dynamic viscosity of the nanofluid relative to the base fluid.

As a function of temperature for Ni/water nanofluids at different nanoparticle concentrations, the results demonstrate a clear trend in which the relative dynamic viscosity increases with both rising temperature and nanoparticle concentration. Among the tested concentrations, the nanofluid with the highest Ni content (S1) exhibits the greatest increase in viscosity, followed by the nanofluid containing S3. The lowest concentration (S2) results in the smallest increase in viscosity. At lower temperatures (293 K), the relative dynamic viscosity increases range from approximately 0.05% to 4.48%, depending on the nanoparticle concentration. As the temperature increases (323 K), the viscosity increases more significantly, with values ranging between 3.8% and 12.6%. This temperature-dependent increase in viscosity highlights the complex interactions between the nanoparticles and the base fluid at different concentrations and temperatures.

**Figure 10:**
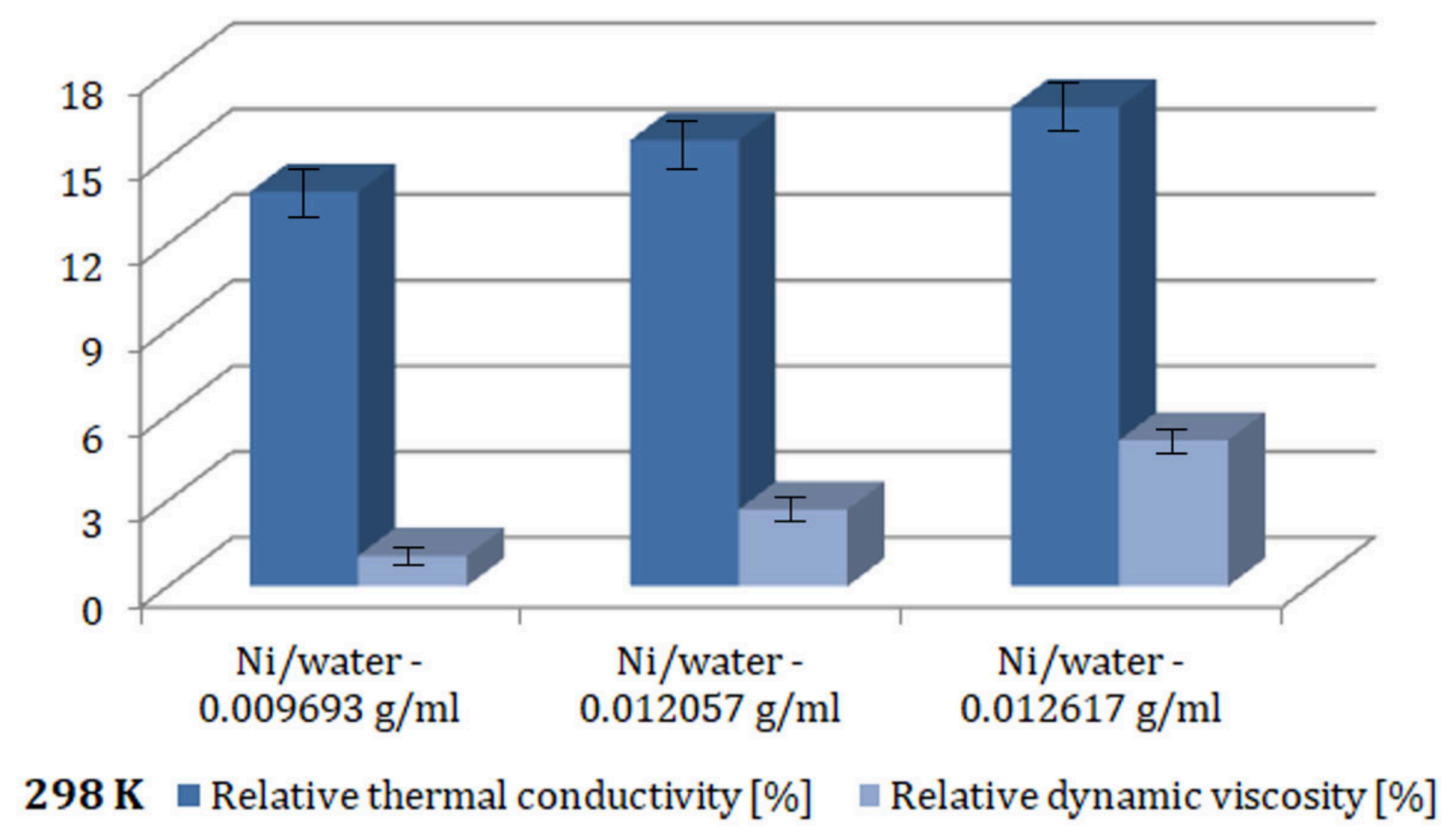
The increase in thermal conductivity and dynamic viscosity at 298 K.

Figure 10 shows the comparative analysis of the relative increase in thermal conductivity and dynamic viscosity at a temperature of 298 K for Ni/water nanofluid at different nanoparticle concentrations. As can be seen, as nanoparticle concentration increases, both thermal conductivity and dynamic viscosity increase. At all the concentrations studied, the increase in thermal conductivity was more significant compared to the increase in dynamic viscosity. The higher increase in thermal conductivity relative to viscosity at higher nanoparticle concentrations suggests that the addition of nanoparticles is enhancing the heat transfer properties of the fluid much more than the increase in viscosity. At the highest concentration (S1), thermal conductivity increases by 16.78%, whereas dynamic viscosity increases by only 5.12%. The results presented in Figure 10 suggest that the S3 sample offers a compromise between maximizing thermal conductivity and maintaining acceptable viscosity levels. These properties make the Ni/water nanofluid a promising candidate for advanced thermal management applications, including electronic cooling systems and automotive cooling circuits.

### 3.3. Photoelectrochemical Characterization of the Ni Nanoparticles

Potentiostatic PEC measurements under chopped illumination highlight the influence of Ni nanoparticle loading on the photoelectrocatalytic behavior of TiO_2_ thin films. The TiO_2_ thin films were produced by Pulsed Laser Deposition just to act as photoactive material support. The TiO_2_ thin films were deposited on Pt/SiO_2_/Si (100) substrates, with the 100 nm thick Pt film acting as bottom electrode for the three-electrode configuration PEC measurements. While both Ni-modified electrodes exhibit more negative photo potentials than bare TiO_2_, consistent with improved electron extraction and enhanced hydrogen-evolution kinetics, the magnitude of this improvement depends strongly on the amount of deposited Ni nanoparticles of the S5 probe on top of the TiO_2_ thin films.

Table 5 summarizes the samples used in PEC experiments:

Sample P8 shows the most pronounced photo potential shift, indicating an optimal balance between catalytic site density and efficient charge transfer at the TiO_2_/Ni interface. In contrast, the P9 sample displays a reduced enhancement, likely due to excessive surface coverage that partially blocks light absorption or introduces additional charge-transfer resistance. This behavior suggests that Ni loading follows a non-linear trend, where moderate nanoparticle coverage maximizes catalytic performance, while overloading can suppress photocatalytic efficiency. Thus, the data reveal that controlled Ni deposition is essential for achieving the optimal synergy between TiO_2_ photoactivity and Ni-mediated hydrogen-generation catalysis.

**Figure 11:**
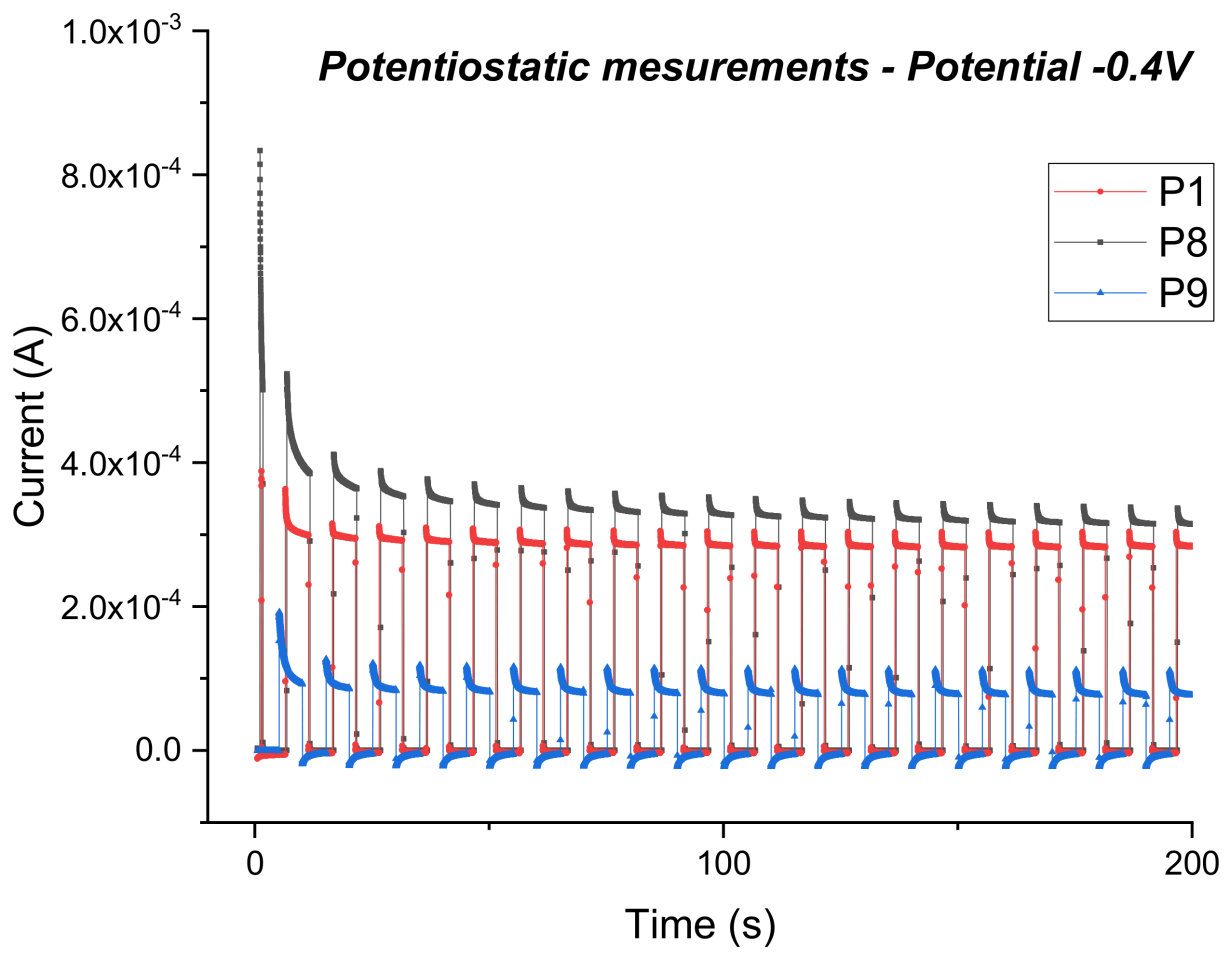
Potentiostatic PEC measurements under chopped irradiation on TiO_2_ samples with/without surface-loaded Ni nanoparticles.

**Figure 12:**
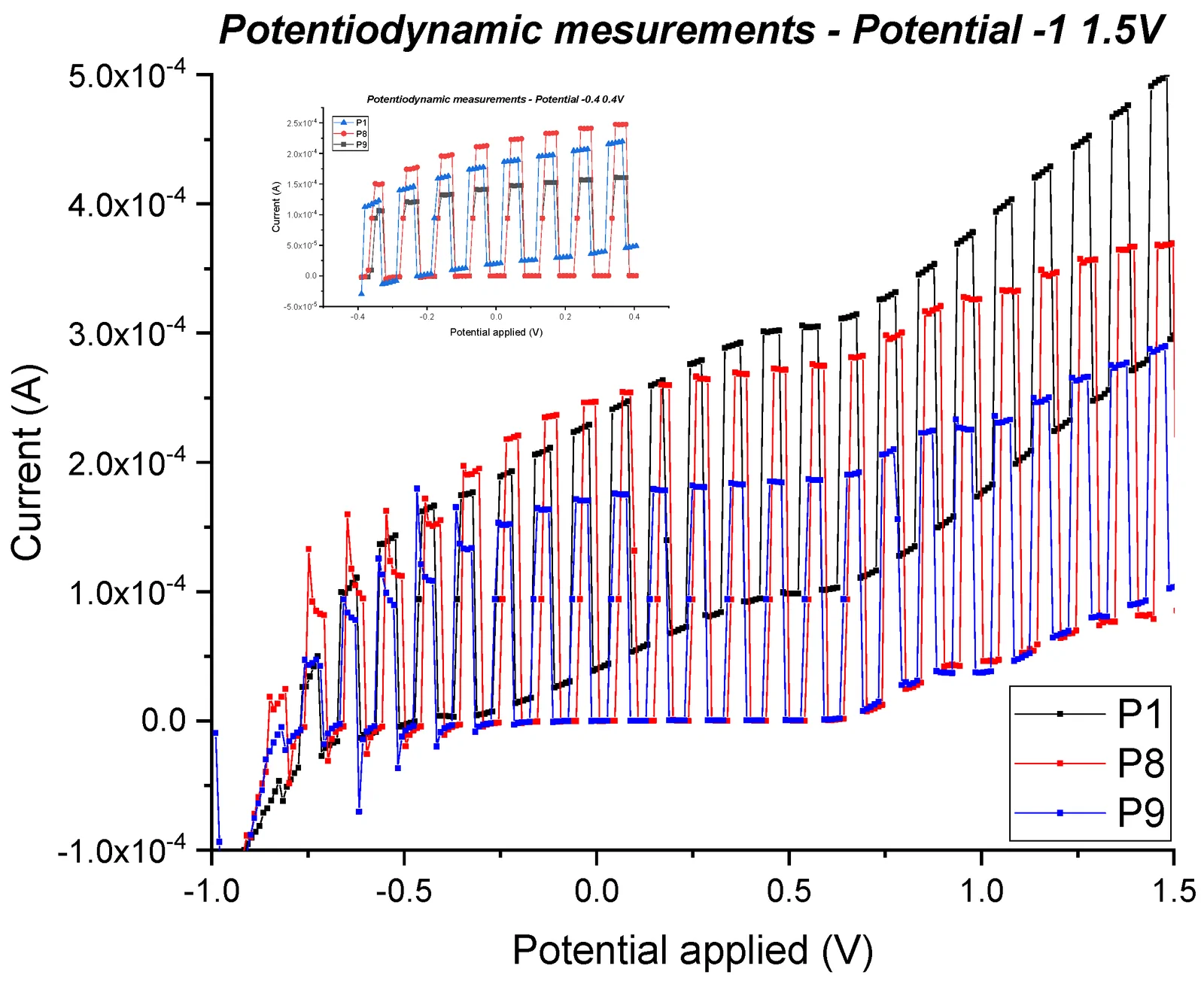
Potentiodynamic PEC measurements under chopped irradiation on TiO_2_ samples with/without surface-loaded Ni nanoparticles.

Potentiodynamic and potentiostatic PEC measurements (Figure 11 and Figure 12) under chopped illumination were recorded in 0.5 M NaOH electrolyte solution (pH = 13.7) to evaluate the PEC response of the heterostructures.

Potentiodynamic measurements performed on the same TiO_2_ electrodes further confirms the Ni-loading-dependent trend observed in the potentiostatic PEC measurements. The bare TiO_2_ sample requires the most negative applied potentials to drive small values of photocathodic current, demonstrating intrinsically slow proton-reduction kinetics and significant charge-transfer limitations at the semiconductor–electrolyte interface.

**Figure 13:**
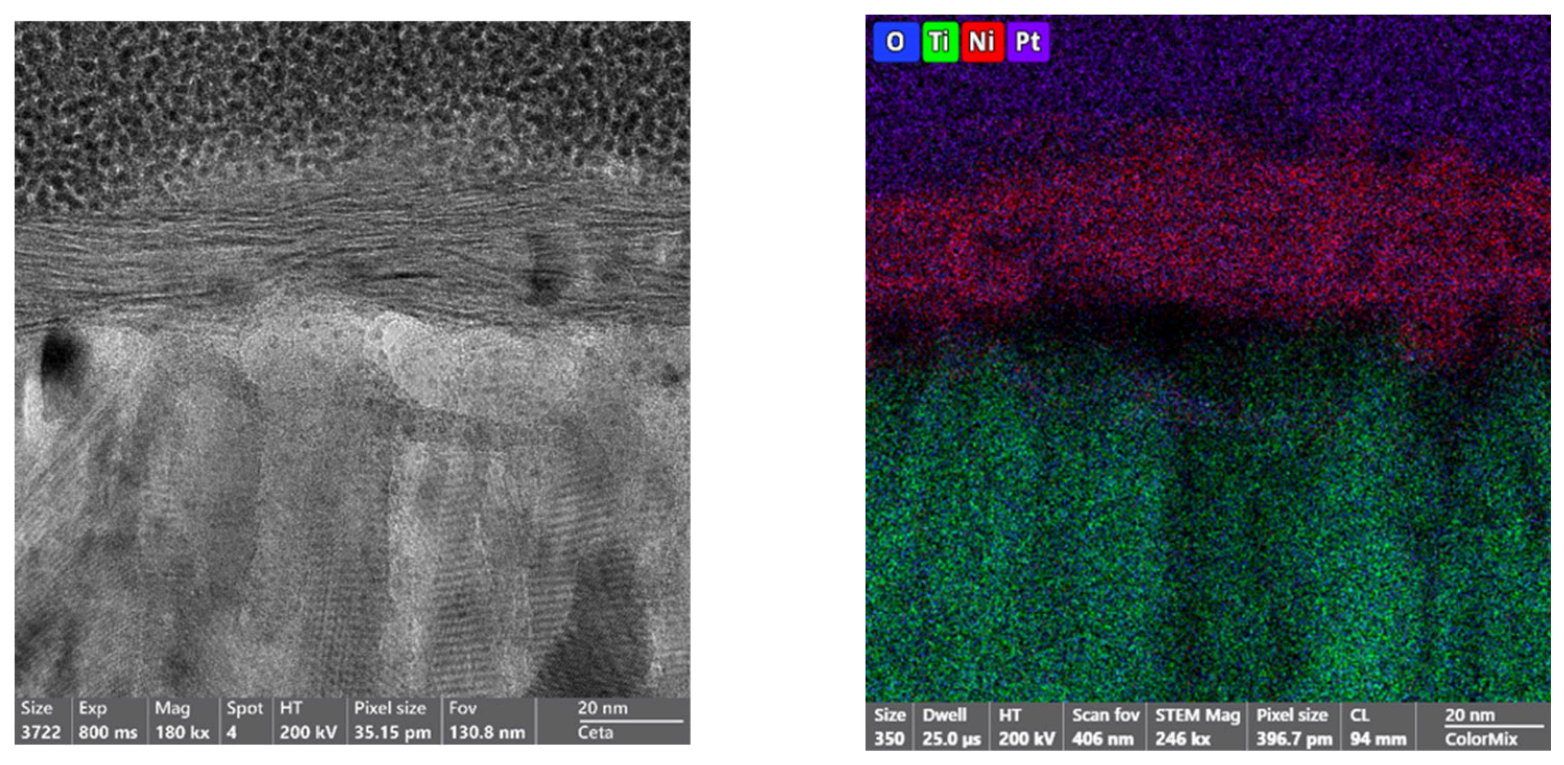
TEM image and STEM EDX mapping in cross-section of Ni-loaded TiO_2_ thin films.

Upon deposition of Ni nanoparticles, the onset potential shifts positively and the photocurrent becomes more pronounced, indicating facilitated electron transfer and improved catalytic activity for hydrogen evolution. However, the magnitude of this enhancement again depends on the Ni loading: the coated electrode (P8 sample) precursor exhibits the most favorable response, showing both a more positive onset potential and higher photocurrent density compared to the P9 sample. The reduced improvement at higher Ni coverage suggests that excessive Ni deposition can lead to partial shielding of the TiO_2_ surface or increased interfacial resistance, thereby limiting light absorption and hindering electron mobility, as can be seen from Figure 13 of the cross-section TEM image and STEM-EDX elemental mapping images of the high-concentration Ni-loaded TiO_2_ thin film surface.

To further study the PEC properties of the TiO_2_/Pt structures functionalized with different amounts of nickel nanoparticles, potentiostatic PEC measurements and potentiodynamic measurements were performed under chopped illumination. The results obtained from the measurements highlight the influence of the applied potential (negative or positive) on the photoanodic and photocathodic behavior of the studied samples.

The potentiostatic PEC measurement at an applied potential of −0.4 V clearly highlights the influence of Ni nanoparticles on the photocathodic response of the TiO_2_/Pt structure. All the studied samples show characteristics of photoelectrochemical processes, with periodic variations in the current associated with the illumination/dark cycles, which confirms the photocatalytic activity of the studied samples.

**Figure 14:**
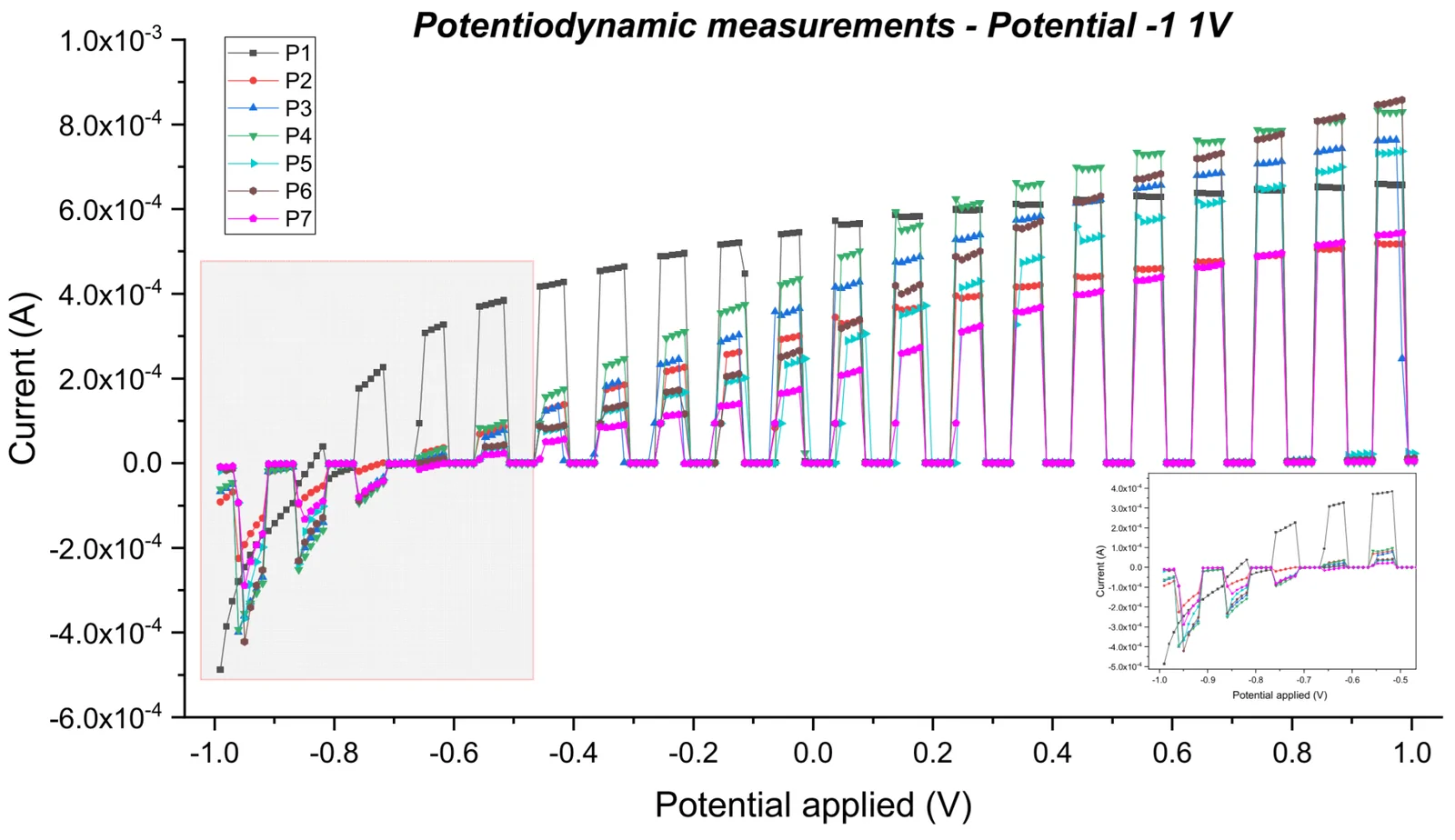
Potentiostatic measurements under chopped illumination of TiO_2_ samples with/without Ni nanoparticles at −0.4 V applied potential.

As can be seen in the graph in Figure 14, the reference sample (TiO_2_/Pt without Ni nanoparticles) shows the highest generated current response, at negative −0.4 V applied potential. The signal shows stable pulses throughout the duration of the experiment, which denotes the electrochemical stability of the system. After the deposition of Ni nanoparticles, a modification of the recorded photoelectrocatalytic response is observed. A gradual decrease in the recorded response is observed, with samples with low amounts of nickel nanoparticles (P2–P4) having a relatively high response (~2 × 10^−4^ A) compared to the reference sample P1 (3.5 × 10^−4^ A). The signals of all samples remain stable throughout the experiment, however the reduction in current indicates that the Ni nanoparticles negatively influence the transport of charge carriers or the absorption of light at negative applied potential.

At higher amounts of nickel nanoparticle suspension (P6, P7), the electrophotocatalytic response decreases significantly, reaching values below 1 × 10^−4^ A and the recorded amplitude of the oscillations becomes visibly smaller, and the negative components of the recorded signal are more pronounced. A tendency of slow decrease in the current overtime can also be observed for all samples, a phenomenon probably associated with the polarization of the electrodes, the accumulation of reaction intermediates or the progressive recombination of charge carriers. However, the general stability of the periodic response indicates a good resistance of the system during the measurements.

The results suggest the existence of an optimal concentration of Ni nanoparticles for enhancing the photoanodic behavior of TiO_2_ thin film. At low concentrations, Ni can facilitate charge separation and electron transfer. From an application point of view, the results confirm that the TiO_2_/Pt architecture exhibits high photoanodic PEC activity, and the modification with Ni nanoparticles must be carefully optimized to avoid the degradation of the PEC performance at negative potential.

**Figure 15:**
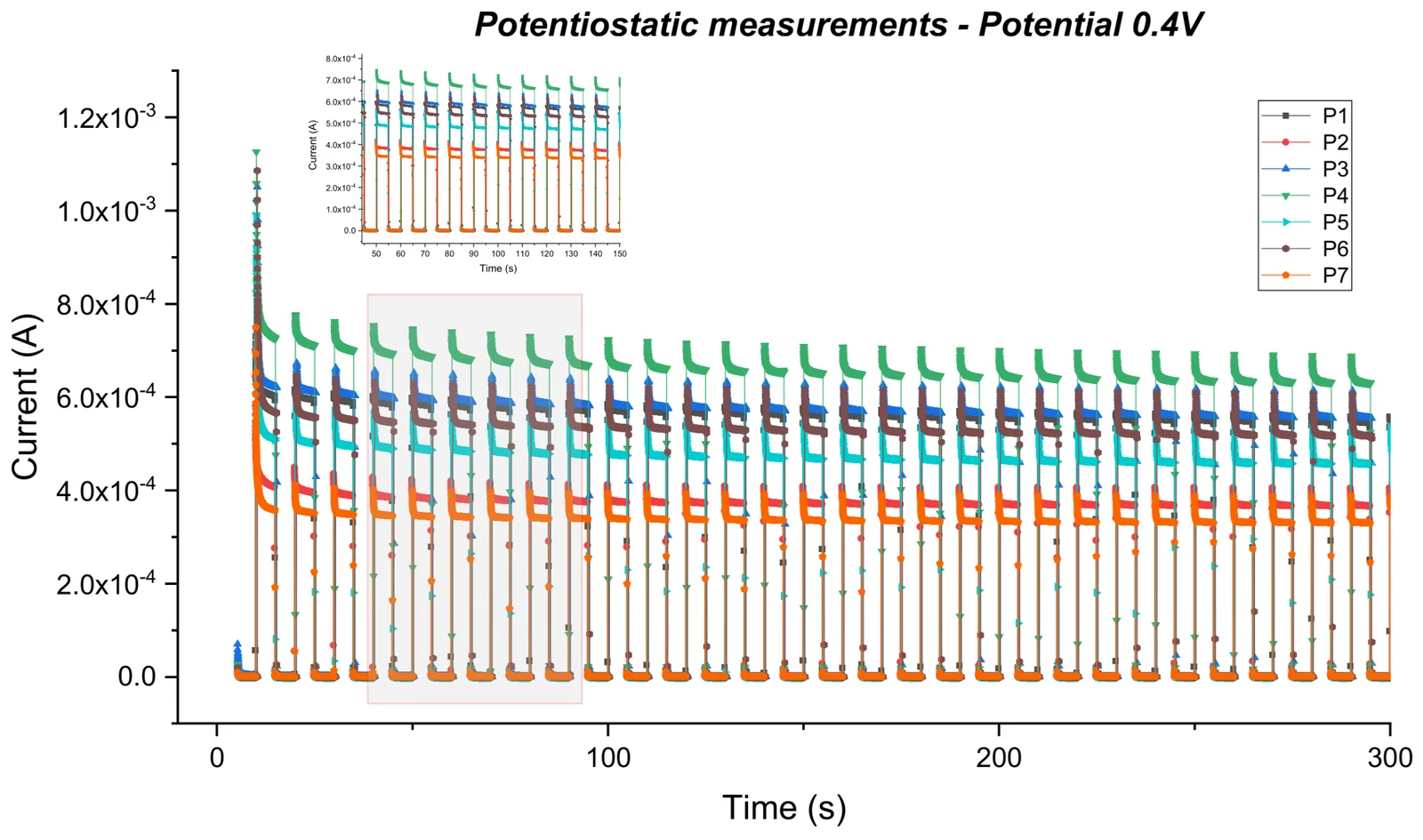
Potentiostatic measurements for TiO_2_ samples with/without loaded Ni nanoparticles at 0.4 V applied potential.

**Figure 16:**
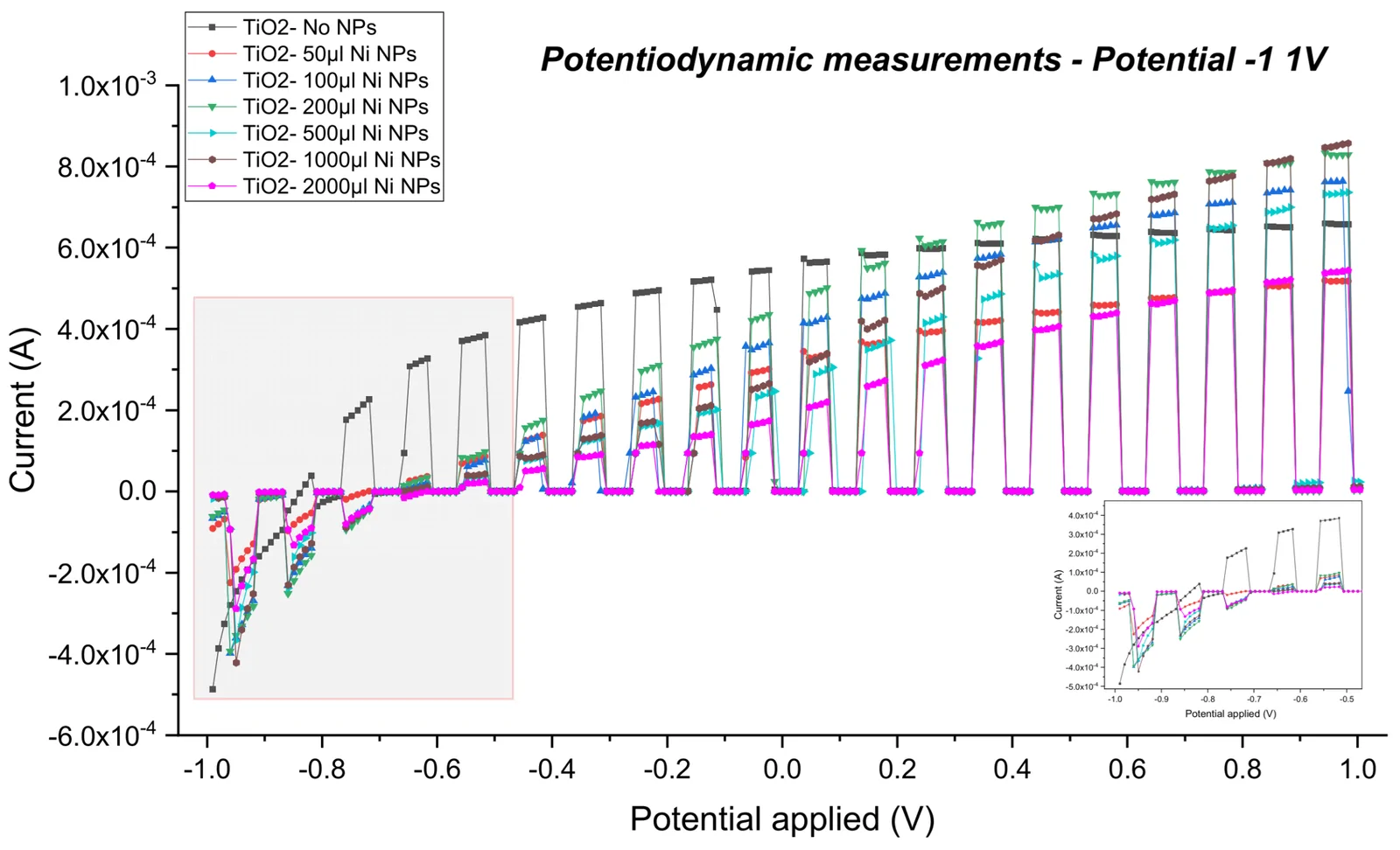
Potentiodynamic measurements of TiO_2_ samples with/without loaded Ni nanoparticles under applied potential. In the inset, the samples’ responses are presented up to −1 V applied potential.

In Figure 15, all samples show regular oscillations of the current associated with successive cycles of illumination and darkness, which confirms the maintenance of the photocatalytic activity of the TiO_2_ layer. Compared to the measurements at negative potential, the current values are significantly higher, reaching the range of 10^−4^–10^−3^ A, which indicates that the anodic polarization favors the charge transfer processes and the separation of photogenerated carriers. Unlike the graph where the results of the photocatalytic activity at negative potentials of −0.4 V are presented where the reference sample has the best electrophotocatalytic response, in the case of the graph shown in Figure 16, the best performances are recorded by the sample P4 of nickel nanoparticle suspension, which reach currents of approximately 6–7 × 10^−4^ A. These results indicate that under positive polarization conditions, nickel nanoparticles contribute favorably to the electron transfer processes, having a cocatalytic effect for electron capture.

For samples with a low amount of nanoparticle suspension, namely P2 and P3, the photoelectrocatalytic response is moderate and improved compared to the reference sample, but the maximum effect appears for intermediate concentrations. This phenomenon indicates the existence of an optimal quantity of nanoparticles suspension for the cocatalytic effect to increase the density of the current produced. At high amounts of nanoparticle suspension (P6, P7), the current begins to decrease progressively, although the recorded values generally remain higher than those observed at negative potential, at −0.4 V. The decrease in the recorded performance is due to the agglomeration of nanoparticles on the surface of the TiO_2_/Pt sample, which reduces light penetration; however, even under these conditions the photocatalytic response of the sample remains stable. Also, the initial current pulses are more intense than in the case of negative polarization, reaching values close to 1.2 × 10^−3^ A.

The potentiodynamic PEC measurements performed in the range −1 V to +1 V (Figure 16) provides important information regarding the photoelectrochemical behavior of the TiO_2_/Pt samples enhanced with Ni nanoparticles. It is observed that all samples show a progressive increase in current with the potential shift towards positive values. The periodic shape of the signal indicates the response of the system to successive illumination cycles, which confirms the photoactive nature of the investigated structures. At each potential step, illumination produces a rapid increase in current, followed by its stabilization in a quasi-stationary regime, characteristic of the separation and transport processes of photogenerated carriers. In the negative potential range (approximately −1 V to −0.6 V), all samples show significant cathodic currents. The sample without Ni nanoparticles has the most pronounced negative response, reaching values of approximately −5 × 10^−4^ A, which indicates a high activity in electrochemical reduction processes. The introduction of Ni nanoparticles reduces the amplitude of the cathodic current.

As the potential becomes less negative and subsequently positive, all samples show an almost linear increase in the anodic current. In this range, a very clear effect of Ni nanoparticles appears; samples with intermediate Ni concentrations (P4–P6) develop the highest photoinduced current values. In particular, sample P4 shows one of the highest anodic current densities over the entire positive potential range, clearly exceeding the sample without Ni. This result suggests that Ni nanoparticles contribute to photocatalysis, acting as a cocatalyst in the process.

At potentials close to +1 V, the anodic currents reach values of approximately 8–8.5 × 10^−4^ A for some Ni-modified samples, while the sample without nanoparticles remains at lower values (~6.5 × 10^−4^ A). This behavior demonstrates that Ni has a pronounced positive effect on the anodic processes.

However, at very high Ni concentrations (P7), a decrease in the electrophotocatalytic performance is again observed. The anodic current becomes lower compared to the samples with a lower amount of nanoparticle suspension, which indicates that the excess of nanoparticles leads to the coverage of the active TiO_2_ surface and the occurrence of agglomeration phenomena on the sample surface.

Comparing these results with potentiostatic PEC measurements at ±0.4 V, a very good agreement between the experimental trends is observed. At negative polarization, Ni nanoparticles reduce the photoelectrochemical activity, while at positive polarization they significantly improve the electrophotocatalytic response, especially for moderate concentrations. LSV measurements thus confirm the existence of an optimal amount of Ni, approximately in the range of 200–500 μL (P4, P5), for which charge transfer and photoelectrocatalytic efficiency are maximal.

## 4. Conclusions

The present study demonstrates that nickel nanoparticles represent a multifunctional application such as improving heat transfer medium and improving photoelectrocatalytic performance. TEM particle characterization showed that synthesized Ni-based nanoparticles exhibit dimensions predominantly in the 5–15 nm range. The probe S2 presented a narrow size distribution, with most particles concentrated between 7 and 9 nm. The calculated standard deviation indicates relatively homogenous particle population with limited variation. From DLS characterization, among all the samples, S1 stands out as the most promising formulation, exhibiting small particle size, uniform size distribution, and high colloidal stability. The thermal conductivity enhancement of Ni nanoparticles was observed; the S1 probe produced the greatest thermal conductivity improvement, representing an enhancement of 20% compared with pure water.

The combined potentiostatic PEC measurements and potentiodynamic measurements provide consistent evidence that the incorporation of Ni nanoparticles significantly improves the photoelectrocatalytic performance of TiO_2_ thin films toward hydrogen evolution, but only within an optimal loading range. Potentiostatic PEC measurements show that both Ni-modified electrodes reach more negative and more stable photo potentials compared to bare TiO_2_, confirming enhanced charge separation and faster electron transfer to the electrolyte. Although the bare TiO_2_ exhibits a pronounced initial peak, this feature reflects transient surface charging rather than efficient catalytic activity. In contrast, Ni-loaded electrodes sustain higher steady-state photocurrents, indicating genuinely improved hydrogen evolution reaction. Potentiodynamic measurements further support this trend: the P8 Ni-loaded sample displays the most positive onset potential and highest photocurrent density, while excessive Ni loading (P9) diminishes performance, likely due to surface coverage, increased resistance, or partial shading that reduces photon absorption. Altogether, these results demonstrate that Ni nanoparticles enhance TiO_2_ photo electrocatalysis by facilitating electron collection and providing active HER sites, but their beneficial effect strongly depends on maintaining an optimal nanoparticle loading that maximizes catalytic activity without compromising light harvesting or interfacial charge transport.

## Figures and Tables

**Table 1:** Comparison of representative nanoparticles used in nanofluids for thermal management.

Nanoparticle	Main Properties	Typical Particle Size	Reference
Ag	High thermal conductivity, antimicrobial activity	60–80 nm	[22]
Cu	High thermal conductivity	<10 nm	[6]
Al_2_O_3_	High chemical stability, good dispersion	<100 nm	[19]
TiO_2_	Chemical stability, photocatalytic activity	21 nm (Degussa P25); <200 nm	[12,13,14]
Ni	Thermal conductivity, magnetic behavior, catalytic activity, electrical conductivity	<400 nm	[10,11,20,21]

**Table 2:** Summary of probes used in the study.

Probe	Material	Wavelength	Pulse Energy	Liquid	Number of Laser Pulses	Concentration
S1	Ni	1064 nm	700 mJ	400 mL	75,000	0.0126 g/mL
S2	Ni	532 nm	350 mJ	400 mL	75,000	0.0096 g/mL
S3	Ni	1064 nm	700 mJ	800 mL	75,000	0.0120 g/mL
S4	Ti	1064 nm	700 mJ	400 mL	75,000	0.0091 g/mL
S5	Ni	1064 nm	460 mJ	800 mL	180,000	0.0006 g/mL

**Table 3:** Particle distribution and TEM characterization.

Probe	TEM Image	Histogram
S1	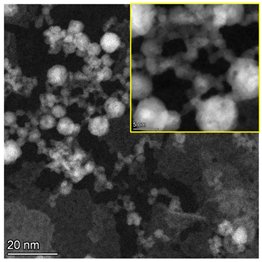	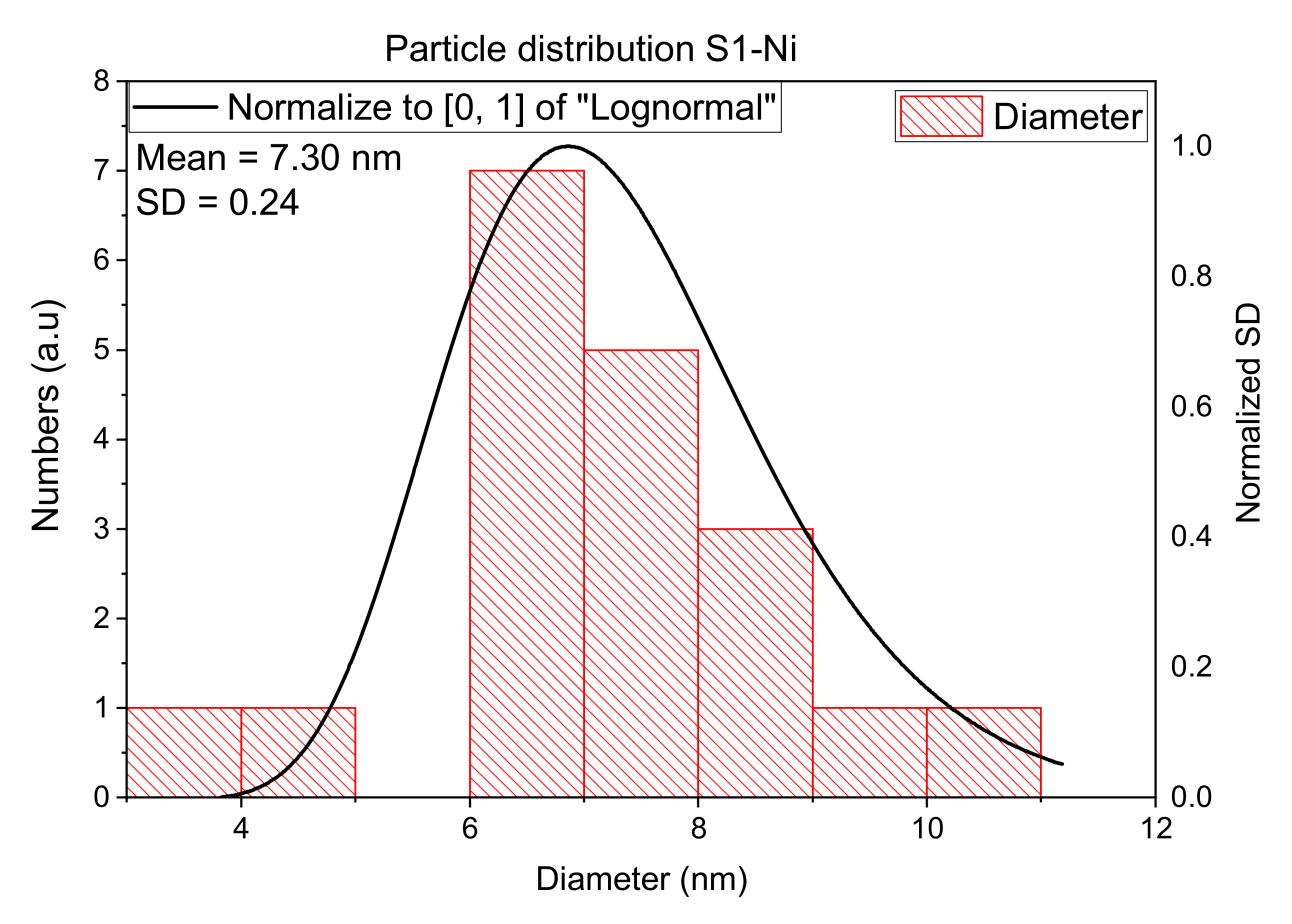
S2	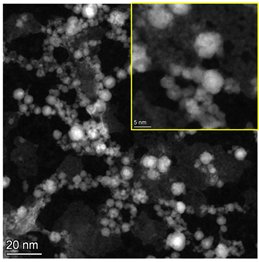	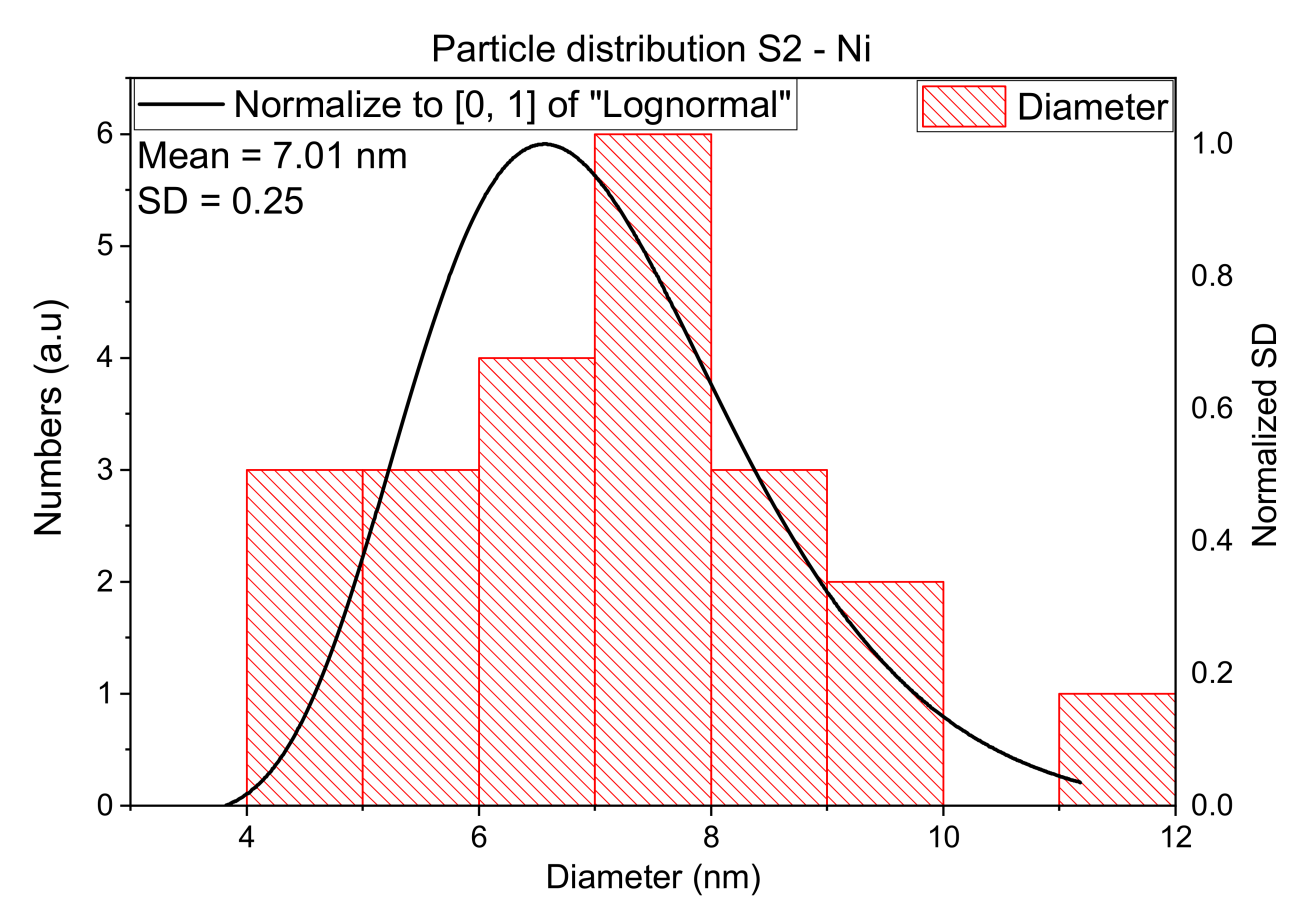
S3	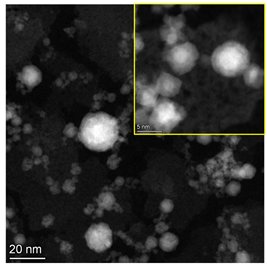	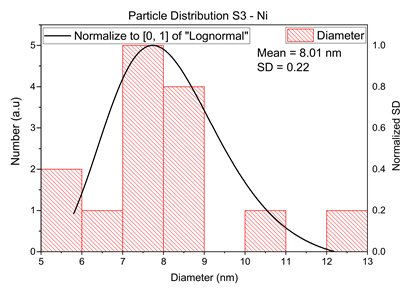
S4	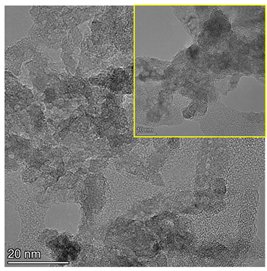	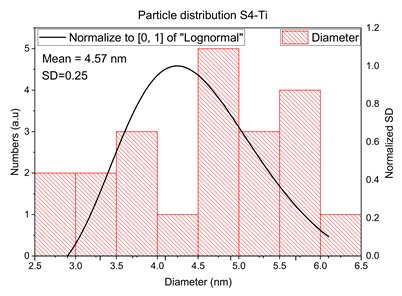
S5	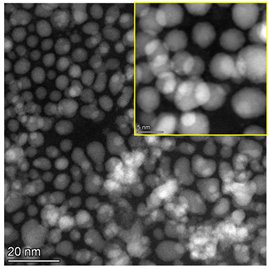	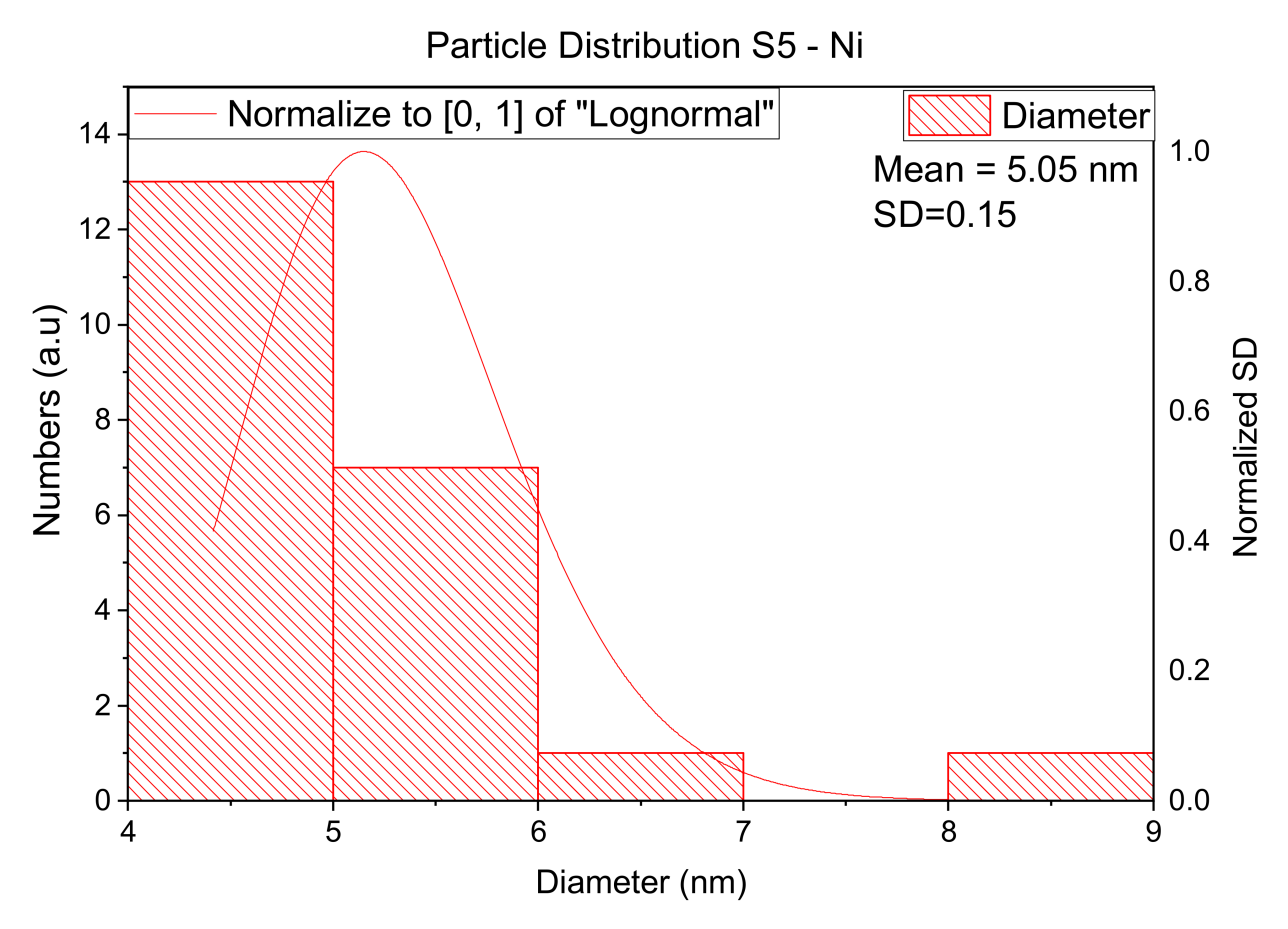

**Table 4:** Interplanar spacing measurements from TEM.

(hkl)	Ref. [34] d (nm)	Calculated for the NiO Nanoparticles
111	0.241	0.257
200	0.209	0.207
220	0.148	0.146

**Table 5:** Summary of probes used in this chapter.

Probe	Deposited Volume (µL)	Pt/Si Substrate
P1	Reference probe	Si/Ti/Pt
P2	50	Si/Ti/Pt
P3	100	Si/Ti/Pt
P4	200	Si/Ti/Pt
P5	500	Si/Ti/Pt
P6	1000	Si/Ti/Pt
P7	2000	Si/Ti/Pt
P8	2100	Si/Ti/Pt
P9	2600	Si/Ti/Pt

## Data Availability

The original contributions presented in this study are included in the article. Further inquiries can be directed to the corresponding author(s).
